# The Deleterious Effects of Tobacco on the Throat and Nose

**Published:** 1888-01

**Authors:** M. F. Coomes

**Affiliations:** Louisville, Ky.


### THE DELETERIOUS EFFECTS OF TOBACCO ON THE THROAT AND NOSE.

Dr. M. F. COOMES, of Louisville, Ky.

He considered smoking far more injurious to these parts than chewing. The smoke came into the mouth heated, and loaded with an irritating oil that would soon coat the mucous membrane, were it not washed away by the saliva. Cigarette smoking is especially injurious, because the smoke is so universally inhaled, causing pharyngitis, laryngitis, and chronic irritation in the nose, not to mention the injury it may occasion to trachea and lungs. Where the smoke is habitually expelled through the nose, we find hypertrophies, congestion, dilated vessels, and a hemorrhagic condition. The smell is impaired or destroyed. The potash salts may also have some effect in adding to the injury. Ninety-five per cent. of smokers have something abnormal or unhealthy about the upper air-passages. In bad cases, he fouud chronic hyperæmia and inflammation of epiglottis with congested cords, and hacking cough to remove the tough mucus; the voice tires easily.—*Columbus Medical Journal.*

